# Microbial Community Differentiation and Predicted Chemical-Defense-Related Functional Potential Across Distinct Microhabitats of Cultured *Hemicentrotus pulcherrimus*

**DOI:** 10.3390/md24070243

**Published:** 2026-07-10

**Authors:** Ding Li, Xiaoping Wu, Fangyu Yuan, Fengfang Zhou, Binxin Cai, Kuncan Wei, Weiqing Huang

**Affiliations:** 1 College of Marine Sciences, Ningde Normal University, Ningde 352100, China; 2 Fujian Provincial University Engineering Research Center for Deep Processing of Mindong Aquatic Products, Ningde 352100, China; 3 School of Medicine, Fuzhou University, Fuzhou 350108, China; 4 College of Biological Science and Engineering, Ningde Normal University, Ningde 352100, China

**Keywords:** *Hemicentrotus pulcherrimus*, microbiome, 16S rRNA gene, predicted functional potential

## Abstract

Sea urchins harbor diverse microbial communities that may contribute to host-associated ecological interactions, microbial competition, and chemical defense. However, the compartment-specific organization of sea urchin-associated microbiota and their predicted chemical-defense-related functional potential remain poorly understood under aquaculture conditions. In this study, 16S rRNA gene amplicon sequencing was used to characterize microbial communities in rearing water, coelomic fluid, intestine, stomach contents, and surface mucus of *Hemicentrotus pulcherrimus* (*H. pulcherrimus*). KEGG Orthology (KO)-based functional prediction was further performed to evaluate predicted chemical-defense-related functional potential, including predicted chemical-defense-related pathways, siderophore-related functions, quorum sensing-related functions, and bacterial competition- and secretion system-related functions. Rarefaction curves and Coverage values indicated sufficient sequencing depth. Alpha diversity and Nonmetric multidimensional scaling (NMDS) analyses revealed clear microbial differentiation among the five sample types, with rearing water showing higher microbial richness. Taxonomic analysis identified *Pseudomonadota*, *Bacteroidota*, *Campylobacterota*, *Bacillota*, *Planctomycetota*, and *Spirochaetota* as dominant phyla, with several discriminative taxa across compartments. KO prediction showed that total predicted abundance of predicted chemical-defense-related KOs differed significantly among sample types. Among host-associated compartments, surface mucus showed relatively higher predicted siderophore-related KO potential, whereas stomach contents showed higher predicted quorum sensing-related KO potential among host-associated compartments. These findings suggest compartment-specific microbial communities and predicted chemical-defense-related functional potential in cultured *H. pulcherrimus* under aquaculture conditions. Because these functions were inferred from 16S-based KO prediction, they should be interpreted as preliminary hypotheses for future metagenomic, metabolomic, and culture-dependent validation.

## 1. Introduction

Sea urchins are important marine invertebrates in coastal ecosystems and aquaculture systems [1,2]. Their body surfaces, digestive tract, and coelomic fluid provide distinct microhabitats for microbial colonization [2,3]. These microbial communities may be involved in nutrient transformation, environmental adaptation, host-associated microbial interactions, and colonization resistance [4]. However, microbial communities associated with different sea urchin compartments are unlikely to be homogeneous because each compartment differs in exposure to the external environment, nutrient availability, host-derived substrates, and potential immune filtering [3].

The digestive tract, stomach contents, surface mucus, and coelomic fluid represent different ecological interfaces between sea urchins and their surrounding environment [2]. Stomach contents may reflect recent dietary and environmental microbial inputs, whereas intestinal microbiota are more closely associated with the host digestive environment [5,6]. Surface mucus directly contacts the surrounding water and may act as an interface for microbial attachment, colonization, and exclusion [7]. Coelomic fluid represents an internal host-associated microenvironment and may be subject to stronger host filtering [3]. Therefore, comparing microbial communities across these compartments can provide insight into microhabitat-specific microbial selection in cultured sea urchins.

Chemical defense is an important ecological strategy in marine organisms and can be mediated by defensive metabolites, microbial competition, chemical signaling, and colonization resistance [8,9]. In marine systems, host-associated microorganisms may contribute to chemical defense through functional traits such as siderophore-mediated iron acquisition, quorum sensing, secretion systems, and bacterial competition, which are closely linked to microbial communication, biofilm formation, population-level responses, and competitive interactions [8,10]. These microbial functions are highly relevant to marine chemical ecology and may provide preliminary clues for future exploration of marine bioactive resources, as marine host-associated microbes have been recognized as promising sources of antimicrobial, antifouling, and other bioactive metabolites [9,11]. Despite growing interest in marine host-associated microbiomes, the potential predicted chemical-defense-related functions of sea urchin-associated microbiota remain poorly characterized, especially under aquaculture conditions. Existing sea urchin microbiome studies have largely focused on microbial diversity, taxonomic composition, coelomic fluid microbiota, gut microbiota, or general predicted metabolic profiles, whereas fewer studies have examined whether different sea urchin microhabitats harbor distinct predicted chemical-defense-related functional profiles [5,12]. In particular, the microbial functional differences among rearing water, coelomic fluid, intestine, stomach contents, and surface mucus remain unclear. It should be noted that 16S rRNA gene amplicon sequencing and PICRUSt2 (version 2.5.2)-based KEGG Orthology (KO) prediction cannot directly verify metabolite production, antibiotic activity, siderophore production, or biosynthetic gene clusters. Therefore, predicted chemical-defense-related functions inferred from such data should be interpreted as predicted functional potential rather than experimentally validated biochemical activity. Nevertheless, this approach can identify candidate microhabitats and microbial groups that deserve further validation through culture-dependent isolation, metagenomics, metabolomics, and compound-level assays.

*Hemicentrotus pulcherrimus* is an economically important sea urchin species in aquaculture, but the organization of its host-associated microbiota and their potential predicted chemical-defense-related functions remain poorly understood [4,13]. In this study, five sample types were collected from cultured *H. pulcherrimus* and their aquaculture environment, including rearing water (AW), coelomic fluid (BCF), intestine (INT), stomach contents (GAS), and surface mucus (TB). Therefore, the results should be interpreted within the context of aquaculture-reared individuals rather than wild or free-living populations. We used 16S rRNA gene amplicon sequencing to investigate microbial communities across these environmental and host-associated compartments, and KO-based functional prediction was further applied to evaluate their predicted chemical-defense-related functional potential. The objectives were to: (a) characterize microbial diversity and community composition across different *H. pulcherrimus* microhabitats under aquaculture conditions; (b) identify shared and compartment-specific microbial taxa; (c) determine differentially abundant bacterial groups among sample types; and (d) evaluate predicted chemical-defense-related functional profiles across rearing water and host-associated compartments.

## 2. Results

### 2.1. Sequencing Depth and Alpha Diversity of Microbial Communities

Rarefaction curves based on the Chao1, Shannon, and Coverage indices gradually approached saturation for all samples, indicating that the sequencing depth was sufficient to capture the major bacterial diversity in rearing water and *H. pulcherrimus*-associated compartments (Supplementary Figure S1). The Coverage curves were close to 1 across the five sample types, further suggesting that the sequencing effort provided adequate Coverage for downstream community comparisons. The five sample types showed distinct alpha diversity patterns. The rearing water group exhibited the highest Chao1 richness, whereas the host-associated compartments displayed lower richness values. Among the *H. pulcherrimus*-associated samples, intestine and coelomic fluid generally showed higher richness than stomach contents, while stomach contents exhibited relatively lower Chao1 values (Supplementary Figure S1). These results indicate that the rearing water contained a broader environmental microbial pool, whereas the microbial communities in *H. pulcherrimus*-associated compartments were more restricted.

Shannon diversity also differed among the five sample types. Rearing water showed the highest Shannon index, suggesting a more diverse and even microbial community in the surrounding aquaculture environment. In contrast, surface mucus showed relatively low Shannon diversity, whereas intestine showed higher diversity among the host-associated compartments. Coelomic fluid and stomach contents showed intermediate Shannon diversity patterns (Supplementary Figure S1). These alpha diversity results indicate that different microhabitats of cultured *H. pulcherrimus* contained distinct microbial richness and evenness under aquaculture conditions, with the external water environment harboring the broader microbial diversity and host-associated compartments showing different degrees of microbial selection.

### 2.2. Microbial Community Structure Differed Significantly Among Sample Types

**Figure 1:**
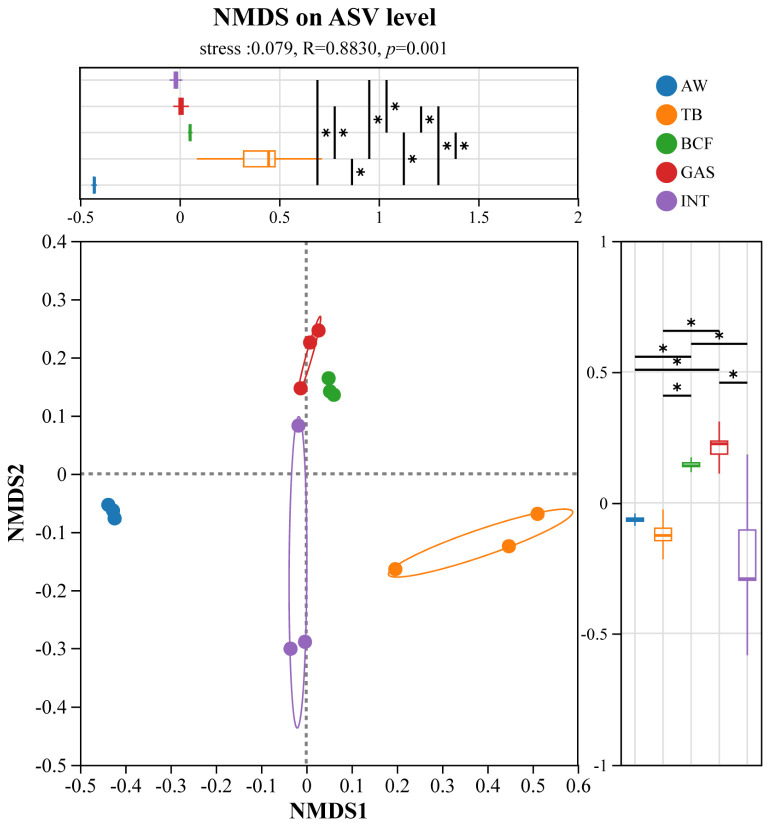
Microbial community differentiation among rearing water and *H. pulcherrimus*-associated compartments based on ASV-level NMDS analysis (AW, rearing water; TB, surface mucus; BCF, coelomic fluid; GAS, stomach contents; INT, intestine). Each sample type contained three biological replicates. For host-associated compartments, each biological replicate represented pooled material from three sea urchins collected within the same sampling area. * *p* < 0.05.

NMDS analysis based on ASV-level community composition revealed clear separation among rearing water, surface mucus, coelomic fluid, stomach contents, and intestine (Figure 1). The low stress value of 0.079 indicated that the ordination reliably represented community dissimilarities among samples. ANOSIM further showed a strong and significant separation among groups, with R = 0.8830 and *p* = 0.001.

The rearing water samples clustered separately from all *H. pulcherrimus*-associated compartments, indicating that the microbial community in the surrounding aquaculture water was distinct from host-associated microbiota. Surface mucus also formed a separate cluster, suggesting that the external mucus layer harbored a microbial assemblage different from both rearing water and internal host-associated compartments. Coelomic fluid, stomach contents, and intestine occupied different positions in the NMDS space, indicating that internal and digestive microhabitats were characterized by distinct microbial community structures (Figure 1). Together, these results show that *H. pulcherrimus*-associated microbiota were strongly structured by sample type rather than representing a uniform host-associated microbial community.

### 2.3. Shared and Compartment-Specific Microbial Members at Different Taxonomic Levels

UpSet analysis showed that the five sample types contained both shared and compartment-specific microbial members. At the ASV level, rearing water contained the largest number of ASVs, with 1402 ASVs detected. The numbers of ASVs detected in surface mucus, intestine, coelomic fluid, and stomach contents were 662, 661, 618, and 433, respectively (Supplementary Figure S2). A total of 144 ASVs were shared by all five sample types, indicating the presence of a common microbial fraction across the aquaculture environment and *H. pulcherrimus*-associated compartments. However, 857 ASVs were unique to rearing water, suggesting that the surrounding water represented a broad environmental microbial reservoir. In contrast, the host-associated compartments contained fewer total and unique ASVs, indicating that *H. pulcherrimus* microhabitats contained more restricted and compartment-selected microbial assemblages.

Similar patterns were observed at higher taxonomic levels. At the phylum level, rearing water contained 38 phyla, while surface mucus, intestine, stomach contents, and coelomic fluid contained 30, 26, 23, and 23 phyla, respectively. Nineteen phyla were shared by all five sample types, indicating that broad taxonomic groups were widely distributed across environmental and host-associated samples (Supplementary Figure S3). However, the differences in total phylum numbers among groups suggest that microbial taxonomic breadth varied across microhabitats. At the class level, 67 classes were detected in rearing water, whereas surface mucus, intestine, coelomic fluid, and stomach contents contained 46, 42, 39, and 36 classes, respectively. This pattern again indicates that rearing water had the highest taxonomic breadth, while host-associated compartments contained a subset of the broader environmental microbial diversity (Supplementary Figure S4). At the family level, rearing water contained 252 families, followed by surface mucus, intestine, coelomic fluid, and stomach contents, with 170, 155, 155, and 118 families, respectively. Eighty families were shared by all five groups, while many families were specific to rearing water (Supplementary Figure S5). At the genus level, rearing water contained 418 genera, while surface mucus, intestine, coelomic fluid, and stomach contents contained 280, 250, 242, and 175 genera, respectively. A total of 112 genera were shared by all groups, and 149 genera were specific to rearing water (Supplementary Figure S6). These results consistently show that rearing water contained the highest microbial taxonomic richness, while *H. pulcherrimus*-associated compartments contained more restricted and compartment-specific microbial assemblages.

### 2.4. Taxonomic Composition of Microbial Communities Across Different Sample Types

**Figure 2:**
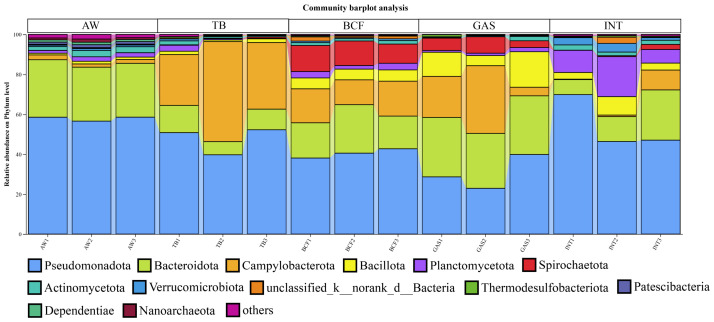
Phylum-level taxonomic composition of microbial communities across different sample types (AW, rearing water; TB, surface mucus; BCF, coelomic fluid; GAS, stomach contents; INT, intestine). Each sample type contained three biological replicates. For host-associated compartments, each biological replicate represented pooled material from three sea urchins collected within the same sampling area.

The taxonomic composition of microbial communities differed markedly among rearing water and *H. pulcherrimus*-associated compartments. At the phylum level, the dominant microbial groups included *Pseudomonadota*, *Bacteroidota*, *Campylobacterota*, *Bacillota*, *Planctomycetota*, *Spirochaetota*, *Actinomycetota*, *Verrucomicrobiota*, *Thermodesulfobacteriota*, *Patescibacteria*, *Dependentiae*, and *Nanoarchaeota* (Figure 2). Among these, *Pseudomonadota* and *Bacteroidota* were widely distributed across all sample types and represented the dominant phyla in most samples. However, their relative abundances varied between rearing water, surface mucus, coelomic fluid, stomach contents, and intestine, indicating compartment-dependent microbial community composition.

At the class level, the major classes included *Gammaproteobacteria*, *Alphaproteobacteria*, *Bacteroidia*, *Campylobacteria*, *Planctomycetes*, *Spirochaetia*, *Clostridia*, *Bacilli*, *Actinobacteria*, and *Verrucomicrobiia* (Supplementary Figure S7). Rearing water was mainly characterized by high proportions of *Gammaproteobacteria*, *Alphaproteobacteria*, and *Bacteroidia*, whereas *H. pulcherrimus*-associated compartments showed more variable class-level compositions. Surface mucus showed a marked contribution of *Campylobacteria* in some samples, whereas intestine contained relatively high proportions of *Gammaproteobacteria*, *Alphaproteobacteria*, *Bacteroidia*, and *Planctomycetes*.

At the order level, the dominant orders included *Rhodobacterales*, *Campylobacterales*, *Enterobacterales*, *Bacteroidales*, *Flavobacteriales*, *Pseudomonadales*, *Spirochaetales*, *Pirellulales*, *Burkholderiales*, and *Peptostreptococcales-Tissierellales* (Supplementary Figure S8). At the family level, *Paracoccaceae*, *Arcobacteraceae*, *Sulfurovaceae*, *Flavobacteriaceae*, *Colwelliaceae*, *Spirochaetaceae*, *Prolixibacteraceae*, *Pirellulaceae*, *Vibrionaceae*, *Marinifilaceae*, *Nitrincolaceae*, *Fusibacteraceae*, *Psychromonadaceae*, *Comamonadaceae*, *Mycobacteriaceae*, *Moraxellaceae*, *Rubritaleaceae*, and *Alteromonadaceae* were the major groups (Supplementary Figure S9). At the genus level, the dominant taxa included unclassified_f_*Paracoccaceae*, *Arcobacter*, *Sulfurovum*, *Colwellia*, *Puteibacter*, *Vibrio*, norank_f_*Marinifilaceae*, *Marinobacterium*, *Fusibacter*, *Psychromonas*, *Mariniblastus*, *Delftia*, and *Mycobacterium* (Supplementary Figure S10).

The distribution of dominant taxa showed clear compartment-specific patterns. *Paracoccaceae* and *Marinobacterium* were more associated with rearing water, whereas *Sulfurovaceae* and *Sulfurovum* were prominent in surface mucus. *Arcobacteraceae*, *Arcobacter*, *Colwelliaceae*, and *Colwellia* showed higher contributions in coelomic fluid and stomach contents. In addition, several digestive-associated samples contained relatively high proportions of *Puteibacter*, *Vibrio*, and norank_f_*Marinifilaceae*. These results indicate that the microbial communities of cultured *H. pulcherrimus* were dominated by marine-associated bacterial lineages, but their taxonomic distributions differed clearly among sample types.

### 2.5. Differentially Abundant Bacterial Taxa Among Sample Types

**Figure 3:**
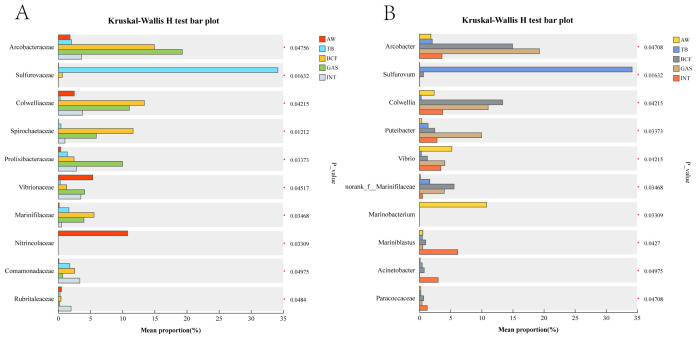
Differentially abundant bacterial taxa among rearing water and *H. pulcherrimus*-associated compartments. (**A**) Differentially abundant bacterial families; (**B**) Differentially abundant bacterial genera (AW, rearing water; TB, surface mucus; BCF, coelomic fluid; GAS, stomach contents; INT, intestine). Each sample type contained three biological replicates. For host-associated compartments, each biological replicate represented pooled material from three sea urchins collected within the same sampling area. * *p* < 0.05.

Kruskal–Wallis tests identified several bacterial taxa that differed significantly among the five sample types (Figure 3). These taxa should be interpreted as compartment-associated candidates rather than definitive biomarkers because the analysis was based on relative abundance data and a limited number of biological replicates. At the family level, *Arcobacteraceae*, *Sulfurovaceae*, *Colwelliaceae*, *Spirochaetaceae*, *Prolixibacteraceae*, *Vibrionaceae*, *Marinifilaceae*, *Nitrincolaceae*, *Comamonadaceae*, and *Rubritaleaceae* showed significant variation among compartments. Correspondingly, at the genus level, *Arcobacter*, *Sulfurovum*, *Colwellia*, *Puteibacter*, *Vibrio*, norank_f_*Marinifilaceae*, *Marinobacterium*, *Mariniblastus*, *Acinetobacter*, and unclassified_f_*Paracoccaceae* differed significantly among groups. Notably, *Arcobacteraceae*/*Arcobacter* and *Colwelliaceae*/*Colwellia* were more abundant in coelomic fluid and stomach contents, whereas *Sulfurovaceae*/*Sulfurovum* were enriched in surface mucus. *Paracoccaceae* and *Marinobacterium* were relatively abundant in rearing water. These results indicate that the microbial community differences among sample types were reflected across both family and genus levels, with specific taxa contributing strongly to compartmental differentiation.

### 2.6. Niche Breadth Patterns and Ecological Strategy Distribution

**Figure 4:**
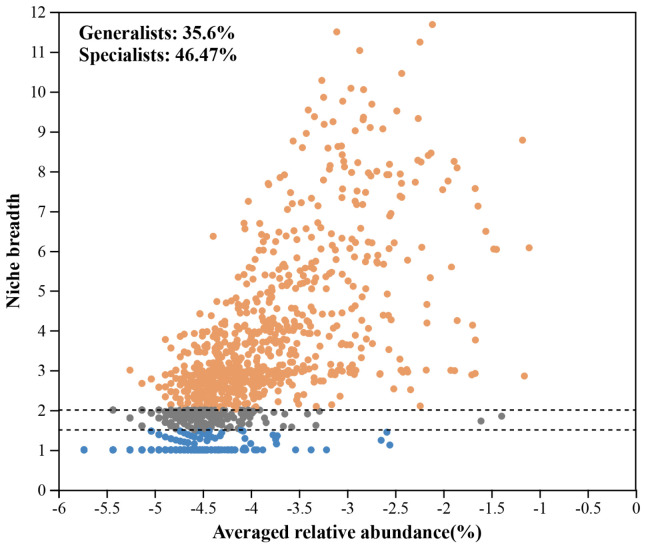
Niche breadth distribution of microbial taxa across rearing water and *H. pulcherrimus*-associated compartments. Each dot represents an individual microbial taxon. Orange dots represent generalists with niche breadth values greater than 2.0, whereas blue dots represent specialists with niche breadth values lower than 1.5. Gray dots represent taxa with intermediate niche breadth values that were not classified as either generalists or specialists. The upper and lower horizontal dashed lines indicate the niche breadth thresholds of 2.0 and 1.5 used to define generalists and specialists, respectively. The percentages shown in the upper-left corner indicate the proportions of generalists and specialists among all analyzed taxa.

Niche breadth analysis showed that microbial taxa differed in their habitat distribution patterns across the five sample types. Specialist taxa accounted for 46.47% of the microbial taxa, whereas generalists accounted for 35.6%. This indicates that specialist taxa represented a larger proportion than generalist taxa in the dataset. The higher proportion of specialist taxa suggests that many microorganisms were restricted to specific sample types rather than being broadly distributed across rearing water and all *H. pulcherrimus*-associated compartments (Figure 4). This result is consistent with the NMDS separation and UpSet patterns, both of which showed that microbial communities were strongly structured by sample type (Figure 1 and Supplementary Figures S2–S6). The presence of generalist taxa also indicates that a subset of microorganisms may persist across multiple microhabitats, including both environmental and host-associated compartments. Therefore, the microbial assemblages of cultured *H. pulcherrimus* appear to be composed of both broadly distributed generalists and compartment-associated specialists, with specialists contributing substantially to microbial differentiation among groups.

### 2.7. Network Topological Roles of Microbial Taxa

**Figure 5:**
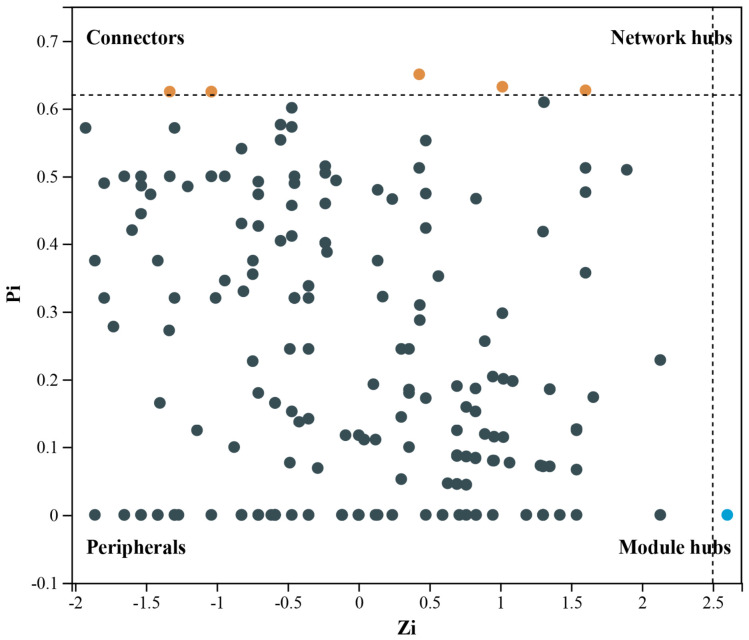
Zi–Pi analysis showing the topological roles of microbial taxa in the co-occurrence network. Each dot represents an individual microbial node. Zi represents within-module connectivity, and Pi represents among-module connectivity. The vertical dashed line at Zi = 2.5 and the horizontal dashed line at Pi = 0.62 indicate the thresholds used to classify nodes into four topological roles: peripherals (Zi ≤ 2.5 and Pi ≤ 0.62), connectors (Zi ≤ 2.5 and Pi > 0.62), module hubs (Zi > 2.5 and Pi ≤ 0.62), and network hubs (Zi > 2.5 and Pi > 0.62). Dark gray dots represent peripheral nodes, orange dots represent connectors, and the blue dot represents a module hub. No network hubs were detected.

Zi-Pi analysis was used to evaluate the potential topological roles of microbial taxa in the co-occurrence network. Most taxa were located in the peripheral region, indicating that the majority of microbial nodes had low within-module connectivity and limited among-module connectivity (Figure 5). A small number of taxa were located in the connector region, suggesting that these taxa may contribute to connections among different network modules. Only very few nodes approached the module hub or network hub regions. This network role distribution indicates that the microbial network was mainly composed of peripheral taxa, while only a limited number of taxa potentially acted as module connectors. This pattern is consistent with the strong compartmental differentiation observed in the community composition results. Because the network was based on correlation analysis, the observed co-occurrence patterns should be interpreted as statistical associations rather than direct ecological interactions. Therefore, the identified connectors or hub-like taxa represent putative topological roles that require further experimental validation.

### 2.8. Predicted Chemical-Defense-Related KO Profiles Across Sample Types

**Figure 6:**
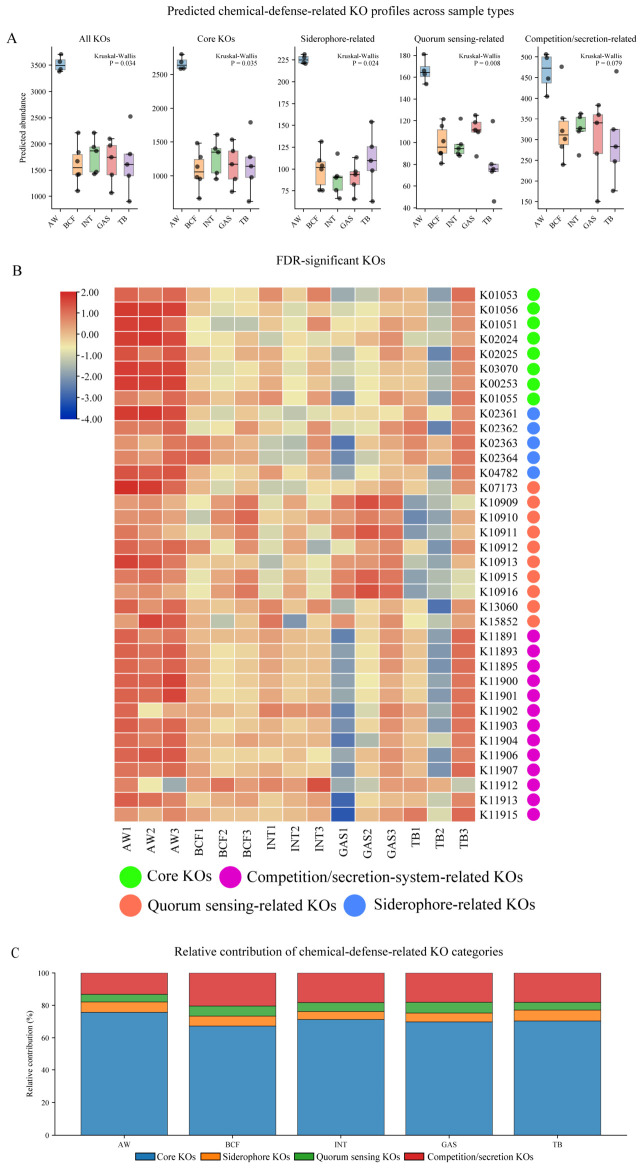
Predicted chemical-defense-related KO profiles across rearing water and *H. pulcherrimus*-associated compartments (AW, rearing water; TB, surface mucus; BCF, coelomic fluid; GAS, stomach contents; INT, intestine). (**A**) Predicted abundance of all predicted chemical-defense-related KOs, core KOs, siderophore-related KOs, quorum sensing-related KOs, and bacterial competition/secretion-related KOs; (**B**) heatmap of FDR-significant KOs based on row-scaled Z-scores, showing compartment-specific variation in core, siderophore-related, and quorum sensing-related KOs; (**C**) relative contribution of the four predicted chemical-defense-related KO categories to the predicted functional profiles of each sample type.

KEGG Orthology (KO)-based functional prediction was performed to evaluate predicted chemical-defense-related functional potential across rearing water and cultured *H. pulcherrimus*-associated compartments under aquaculture conditions (Figure 6). The weighted nearest sequenced taxon index (NSTI) values ranged from 0.038 to 0.109, with an overall mean value of 0.065 ± 0.026 across all samples. Mean NSTI values were 0.047 ± 0.004 for AW, 0.101 ± 0.011 for BCF, 0.081 ± 0.002 for GAS, 0.047 ± 0.008 for INT, and 0.047 ± 0.009 for TB. These relatively low NSTI values indicate that the ASVs were generally well represented by reference genomes and support the reliability of the PICRUSt2-based functional prediction (Supplementary Table S4).

The total predicted abundance of chemical-defense-related KOs differed significantly among the five sample types. Rearing water showed the highest mean predicted KO abundance; however, this pattern should not be interpreted as evidence that rearing water contributes directly to *H. pulcherrimus* holobiont chemical defense, because rearing water represents the surrounding environmental microbial pool rather than a host-associated compartment. Instead, the higher predicted KO abundance in rearing water may largely reflect its higher microbial richness and broader free-living microbial diversity. Therefore, host-associated interpretations were focused primarily on coelomic fluid, intestine, stomach contents, and surface mucus.

Predicted chemical-defense-related KOs also differed significantly among groups. The highest mean abundance was observed in rearing water, followed by intestine, surface mucus, stomach contents, and coelomic fluid. At the individual KO level, two core predicted chemical-defense-related KOs, K01051 and K02024, remained significant after FDR correction. These results suggest that core predicted chemical-defense-related functional potential was mainly associated with rearing water, whereas intestine showed the highest predicted abundance among host-associated compartments.

Siderophore-related KOs showed significant differences among the five sample types. Rearing water again showed the highest mean abundance, followed by surface mucus, coelomic fluid, stomach contents, and intestine. Among the five siderophore-related KOs, K02361 and K02364 remained significant after FDR correction. Notably, surface mucus showed the highest siderophore-related predicted abundance among *H. pulcherrimus*-associated compartments, suggesting that iron acquisition-related microbial functional potential may be more pronounced at the host–environment interface.

Quorum sensing-related KOs showed the strongest group-level differentiation among the functional categories. The mean predicted abundance was highest in rearing water, followed by stomach contents, coelomic fluid, intestine, and surface mucus. Eight quorum sensing-related KOs remained significant after FDR correction, including K07173, K10909, K10910, K10911, K10913, K10915, K10916, and K13060. Among the host-associated compartments, stomach contents showed the highest quorum sensing-related predicted abundance, indicating that microbial communication-related functional potential may be relatively more prominent in this digestive microhabitat.

In contrast, bacterial competition and secretion system-related KOs did not differ significantly among the five sample types at the category level. Although rearing water showed the highest mean abundance, followed by coelomic fluid, intestine, stomach contents, and surface mucus, the group-level difference did not reach statistical significance. At the individual KO level, several KOs showed nominal differences based on raw *p* values, but none remained significant after FDR correction. Therefore, the current dataset does not provide strong evidence for compartment-specific differentiation of bacterial competition and secretion system-related predicted functional potential.

Overall, the KO prediction results showed that predicted chemical-defense-related functional potential varied among sample types in a category-dependent manner. Rearing water had the highest predicted abundance for total predicted chemical-defense-related KOs, core predicted chemical-defense-related KOs, siderophore-related KOs, and quorum sensing-related KOs, consistent with its higher microbial richness. Among *H. pulcherrimus*-associated compartments, intestine showed relatively higher core predicted chemical-defense-related potential, surface mucus showed relatively higher siderophore-related potential, and stomach contents showed relatively higher quorum sensing-related potential. These results suggest that different *H. pulcherrimus* microhabitats may harbor distinct predicted chemical-defense-related functional profiles.

## 3. Discussion

### 3.1. Compartment-Specific Microbial Assembly in Cultured H. pulcherrimus

This study revealed clear microbial differentiation among rearing water, coelomic fluid, intestine, stomach contents, and surface mucus (TB) of cultured *H. pulcherrimus*. NMDS analysis showed significant separation among the five sample types, indicating that microbial community structures were strongly shaped by microhabitat type rather than being uniformly distributed across the aquaculture environment and host-associated compartments (Figure 1). This pattern is consistent with previous sea urchin microbiome studies showing that gut-associated microbial communities differ from surrounding seawater, diet-associated microbiota, and other digestive compartments, supporting the concept that sea urchin-associated microbial assemblages are selectively structured by host-related conditions [4,14].

This interpretation is further supported by niche breadth analysis, in which specialist taxa accounted for 46.47% of the microbial taxa, exceeding the proportion of generalists (Figure 4). The higher proportion of specialists suggests that many microorganisms were restricted to particular compartments rather than being widely distributed across water and all host-associated samples. Similar host-associated selection has been described in natural and laboratory aquaculture sea urchins, where gut microbial communities differed in composition, co-occurrence patterns, and predicted functional attributes depending on habitat, diet, and rearing conditions [6,14].

### 3.2. Digestive Microbiota Reflect Both Environmental Input and Host-Associated Selection

The intestine and stomach contents represented two digestive microhabitats with distinct microbial features. In the present study, stomach contents had fewer ASVs and genera than the intestine, and the two compartments occupied different positions in the NMDS ordination (Figure 2 and Supplementary Figure S1). This suggests that microbial communities changed within the digestive system rather than being uniform along the digestive tract. Similar compartmentalization of sea urchin digestive microbiota has been reported in *Strongylocentrotus purpuratus*, where different gut regions showed distinct bacterial communities, predicted functional attributes, and co-occurrence patterns [6,15]. The stomach contents may be more strongly affected by recently ingested material and transient microbes, whereas the intestine may represent a more stable host-associated digestive microenvironment. This interpretation is also consistent with studies showing that gut digesta and gut tissues of *Lytechinus variegatus* differ in microbial composition and predicted functions under natural and laboratory aquaculture conditions [6].

Previous studies have emphasized the importance of sea urchin gut microbiota in digestive physiology and nutrient processing. For example, the gut microbiome of naturally occurring *Lytechinus variegatus* showed selective microbial attributes and predicted metabolic profiles distinct from seawater and natural diet sources, suggesting that the digestive tract represents a selectively structured microbial habitat [4]. In addition, gut digesta-associated bacteria from five tropical sea urchin species were shown to contain distinct microbial communities and predicted metabolic profiles, further supporting the role of sea urchin gut microbiota in nutrient transformation and host-associated digestion [16]. Diet-dependent gut microbial plasticity has also been reported in sea urchins, indicating that dietary input can reshape intestinal bacterial communities and their functional potential [17].

Several dominant or differentially abundant taxa identified in this study may be related to digestive microhabitat differentiation. For example, *Arcobacteraceae*/*Arcobacter* and *Colwelliaceae*/*Colwellia* were more abundant in coelomic fluid and stomach contents, whereas *Puteibacter* and norank_f_*Marinifilaceae* showed higher contributions in digestive-associated samples. The differential distribution of these taxa suggests that stomach contents and intestine provide distinct microbial niches potentially associated with nutrient availability, organic matter transformation, and microbial interactions within the digestive tract. Similar associations between sea urchin gut microbiota and predicted metabolic potential have been reported in natural *L. variegatus* and other sea urchin species, supporting the view that digestive microbiota may contribute to host-associated nutrient processing and microbial interaction networks [4,17]. However, because the current study used 16S rRNA gene sequencing, these functional interpretations should be treated as ecological hypotheses.

### 3.3. Surface Mucus and Coelomic Fluid Harbored Distinct Host-Associated Microbial Community Structures

Surface mucus and coelomic fluid showed microbial profiles distinct from both rearing water and digestive compartments, suggesting that these two compartments represent different host-associated interfaces [2]. Surface mucus is directly exposed to the surrounding water and therefore represents the first contact zone between sea urchins and environmental microorganisms. Similar surface-associated microbial differentiation has been reported in purple sea urchins, where disease status was associated with marked shifts in the surface microbiome, indicating that the external surface microbiota is responsive to host condition and environmental microbial pressure [7]. In our study, *Sulfurovaceae*/*Sulfurovum* was enriched in surface mucus, while the overall community composition of surface mucus differed from rearing water (Figure 3). Members of *Sulfurovum* are sulfur-oxidizing *Campylobacterota* that commonly occur in chemically dynamic marine environments, including sediments, vent-influenced habitats, and coastal environments [18,19]. Therefore, the enrichment of *Sulfurovaceae*/*Sulfurovum* in surface mucus suggests that the mucus layer may selectively retain specific marine bacterial taxa rather than simply reflecting the surrounding water microbiota. Coelomic fluid, by contrast, represents an internal host-associated interface, and previous work on *Paracentrotus lividus* has shown that coelomic fluid contains a distinct bacterial microbiota with geographic variation, supporting the view that this internal compartment can harbor selected microbial assemblages rather than being microbiologically homogeneous [3].

The coelomic fluid represents an internal host-associated environment with strong physiological and immunological relevance. Genome-level studies of *Strongylocentrotus purpuratus* have revealed an unusually complex innate immune system in sea urchins, including expanded immune receptor repertoires and diverse immune regulators and effectors [20,21,22]. Because coelomocytes are suspended in the coelomic fluid and function as major cellular effectors of sea urchin innate immunity, this internal fluid is expected to represent a more selectively controlled microbial habitat than external surfaces or digestive contents [23]. Direct evidence from *Paracentrotus lividus* further demonstrated that coelomic fluid contains a distinct bacterial microbiota with geographic variation, supporting the view that this compartment is not microbiologically homogeneous or simply contaminated by seawater [3]. Our results are consistent with this framework, as coelomic fluid harbored a microbial community distinct from the other compartments in the NMDS ordination, with several differentially abundant taxa contributing to its compartment-specific profile (Figure 1 and Figure 3). The presence of a specific coelomic fluid-associated microbial assemblage suggests that this internal fluid may represent a constrained microbial habitat shaped by host-associated physiological and immune filtering.

However, the current study did not measure coelomocyte activity, antimicrobial peptide expression, lysozyme activity, or oxidative stress indicators. Therefore, although the distinct microbiota of coelomic fluid may be relevant to host internal homeostasis, it cannot be directly interpreted as evidence of immune function. Future studies should combine coelomic fluid microbiome profiling with immune and physiological indicators to clarify whether specific bacterial taxa are associated with sea urchin health status.

### 3.4. Differentially Abundant Taxa as Candidate Microbial Indicators Across H. pulcherrimus Microhabitats

The compartment-specific distribution of dominant and differentially abundant taxa provides taxonomic evidence for microbial differentiation across cultured *H. pulcherrimus* microhabitats. In this study, several bacterial families and genera, including *Arcobacteraceae*/*Arcobacter*, *Sulfurovaceae*/*Sulfurovum*, *Colwelliaceae*/*Colwellia*, *Vibrionaceae*/*Vibrio*, *Marinifilaceae*, *Marinobacterium*, *Mariniblastus*, *Acinetobacter*, and *Puteibacter*, differed significantly among rearing water, coelomic fluid, the intestine, stomach contents, and surface mucus (Figure 3 and Supplementary Figures S9 and S10). These results indicate that specific bacterial taxa were associated with different host-related or environmental compartments. However, the present 16S rRNA gene dataset does not directly demonstrate the physiological activities, metabolic functions, or chemical-defense roles of these taxa. Therefore, these taxa should be interpreted as candidate microbial indicators of compartmental differentiation rather than confirmed functional contributors.

In the present study, *Sulfurovaceae*/*Sulfurovum* was enriched in surface mucus, indicating its association with this external host–environment interface. Members of *Sulfurovum* belong to *Campylobacterota* and have been widely reported as sulfur-oxidizing bacteria in marine systems. In hydrothermal vent ecosystems, sulfur oxidation is a major driver of microbial primary production, and *Sulfurovum*-related sulfur-oxidizing bacteria are mainly attached to surfaces exposed to diffuse venting, indicating their adaptation to chemically dynamic and surface-associated niches [19,24]. Although sea urchin surface mucus is not comparable to hydrothermal vent environments, these previous studies provide useful ecological context for interpreting the enrichment of *Sulfurovum* in surface mucus. However, sulfur metabolism, sulfur oxidation activity, or chemical-defense activity was not directly measured in this study. Therefore, the enrichment of *Sulfurovum* should be interpreted as a taxonomic pattern that generates hypotheses for future functional validation rather than as direct evidence that sulfur metabolism contributes to chemical defense in *H. pulcherrimus*.

*Arcobacteraceae*/*Arcobacter* showed higher relative abundance in internal and digestive compartments. In the present dataset, this pattern indicates that *Arcobacter* was associated with coelomic fluid and stomach contents. Recent genome-resolved ocean-scale work suggests that *Arcobacteraceae* are widespread mixotrophic bacteria with potential roles in carbon, nitrogen, and sulfur cycling in global oceans [25]. Their enrichment in internal and digestive compartments of *H. pulcherrimus* may therefore reflect adaptation to microhabitats where organic substrates, oxygen gradients, and host-derived compounds coexist. Rather than interpreting *Arcobacter* only as a potential opportunistic bacterium, its compartment-specific distribution may also indicate ecological specialization within sea urchin-associated niches. However, the present study did not directly measure carbon, nitrogen, or sulfur metabolism. Therefore, the compartment-specific distribution of *Arcobacter* should be interpreted as a literature-supported hypothesis of ecological specialization, rather than direct evidence of its functional role in *H. pulcherrimus*-associated microhabitats.

The presence and differential distribution of *Colwelliaceae*/*Colwellia*, *Marinifilaceae*, and *Puteibacter* further indicate that sea urchin-associated compartments contained distinct marine bacterial lineages (Figure 3; Supplementary Figures S9 and S10). Marine *Bacteroidota* are major degraders of algal and animal-derived organic matter, and genome-resolved studies have shown that a limited number of *Bacteroidetes* clades can mediate a large fraction of glycan degradation during algal blooms [26]. *Marinifilaceae* bacteria have also been identified as metabolically versatile contributors to organic matter mineralization in global deep seas [27]. For *Colwellia*, comparative genomic and proteomic studies have shown adaptation to marine organic substrates and environmentally variable conditions, supporting their roles as metabolically flexible marine heterotrophs [28,29]. These previous studies suggest that related taxa may have the capacity to respond to organic substrates in marine environments. Nevertheless, organic matter degradation, substrate utilization, and metabolic activity were not directly tested here. Thus, the higher contributions of these taxa in stomach contents and coelomic fluid should be regarded as compartment-associated taxonomic patterns that require further functional verification.

*Vibrionaceae*/*Vibrio* and *Acinetobacter* also differed significantly among compartments (Figure 3; Supplementary Figures S9 and S10). In the present dataset, these taxa should primarily be viewed as bacterial groups contributing to taxonomic separation among sample types. *Vibrio* species are classical models for quorum sensing-mediated bacterial communication, and quorum sensing regulates collective behaviors such as biofilm formation, virulence-associated traits, secretion systems, and host-associated interactions [30,31,32]. Quorum sensing is also closely linked to secretion systems and microbial competition, providing a mechanistic connection between bacterial communication and competitive colonization [10]. In *Acinetobacter*, quorum sensing has been linked to surface-associated motility and biofilm formation, suggesting that this genus may also contribute to surface colonization and microbial interaction processes [33]. These functional traits are particularly relevant because quorum sensing-related KOs showed the strongest group-level differentiation among the predicted chemical-defense-related categories in this study. However, quorum sensing activity, secretion-system activity, microbial competition, virulence potential, or pathogenicity was not directly detected in this study. Therefore, the occurrence of *Vibrio* and *Acinetobacter* in *H. pulcherrimus*-associated compartments should be interpreted cautiously as candidate indicators for future health-related and functional studies, rather than as direct evidence of chemical-defense activity or disease risk.

The relatively higher abundance of *Marinobacterium* in rearing water also provides a taxonomic pattern relevant to the predicted functional framework, although it should not be overinterpreted as equivalent to *Marinobacter*. Marine bacteria related to the *Marinobacter* lineage are well known for siderophore-mediated iron acquisition, and marine microbial siderophores are structurally diverse natural products with important ecological and biomedical relevance [34,35]. In the present study, siderophore production and iron competition were not directly measured. Therefore, the higher siderophore-related KO abundance observed in surface mucus among host-associated samples should be interpreted as predicted siderophore-related functional potential rather than direct evidence of siderophore production or iron-mediated chemical-defense activity. The combined taxonomic and KO prediction results suggest that iron-mediated microbial interactions may represent a testable hypothesis for future studies of the sea urchin surface mucus microbiome.

Overall, the differentially abundant taxa identified in this study should be regarded as candidate microbial indicators associated with compartmental differentiation rather than confirmed ecological or chemical-defense-related functional contributors. Their potential roles in sulfur metabolism, organic matter transformation, microbial communication, siderophore production, microbial competition, host-associated colonization, or aquaculture health require further validation through metagenomics, metatranscriptomics, metabolomics, cultivation, siderophore assays, antimicrobial screening, and functional experiments.

### 3.5. Predicted Chemical-Defense-Related Functions Reveal a Functional Dimension of H. pulcherrimus-Associated Microbiota

The KO-based prediction provided a preliminary functional perspective on the taxonomic differentiation of *H. pulcherrimus*-associated microbiota. While the 16S rRNA gene analysis showed strong compartmental differentiation, the chemical-defense-related KO profiles suggested that different microhabitats may harbor distinct predicted functional potentials. In this study, total chemical-defense-related KOs, core chemical-defense-related KOs, siderophore-related KOs, and quorum sensing-related KOs differed significantly among rearing water, coelomic fluid, intestine, stomach contents, and surface mucus (Figure 6). However, these KO profiles were inferred from 16S rRNA gene-based PICRUSt2 prediction and should therefore be interpreted as predicted functional potentials rather than direct evidence of gene abundance, gene expression, or biochemical activity.

The highest predicted abundance of chemical-defense-related KOs occurred in rearing water. This pattern may be partly explained by the substantially higher microbial richness and larger number of unique ASVs observed in the AW group (Figure 6). At the ASV level, rearing water contained 1402 ASVs and 857 AW-specific ASVs, far exceeding the *H. pulcherrimus*-associated compartments (Supplementary Figure S2). Therefore, the higher predicted KO abundance in AW should not be interpreted as direct evidence of stronger chemical-defense activity or true functional enrichment. Instead, it may largely reflect the broader microbial diversity and larger environmental microbial pool present in the aquaculture water. In aquaculture ecosystems, surrounding water is increasingly recognized as a microbial reservoir that interacts with host-associated microbiota and contributes to host–environment microbial exchange [36]. Therefore, in the present aquaculture system, AW may represent a broad microbial and predicted functional reservoir interacting with sea urchin surfaces, digestive contents, and internal compartments. However, whether these predicted functions are actively expressed, retained, or filtered by host-associated microhabitats requires further validation using metagenomic, metatranscriptomic, metabolomic, and culture-based approaches.

More importantly, the host-associated compartments displayed distinct predicted functional tendencies rather than identical predicted chemical-defense-related profiles. Among the host-associated compartments, intestine had the highest core predicted chemical-defense-related KO abundance, surface mucus had the highest siderophore-related potential, and stomach contents had the highest quorum sensing-related potential. Spatially structured marine host-associated microbiomes are widely recognized to reflect microhabitat-specific selection, dispersal limitation, and host filtering, which can lead to compartment-specific taxonomic and functional patterns [36]. Such predicted functional patterns are particularly relevant to marine chemical ecology because host-associated microorganisms are increasingly recognized as reservoirs of bioactive natural products and chemically mediated interaction traits [37,38].

However, these findings should be interpreted cautiously because KO-based functional prediction does not directly demonstrate siderophore production, antibiotic biosynthesis, or predicted chemical-defense activity. The enrichment of siderophore-related and quorum sensing-related KOs identifies surface mucus and stomach contents as candidate compartments for future validation rather than confirming their direct biochemical function.

### 3.6. Compartment-Specific Predicted Chemical-Defense-Related Potentials in Cultured H. pulcherrimus

Predicted chemical-defense-related functions exhibited clear compartment-specific patterns across cultured *H. pulcherrimus* microhabitats. Because PICRUSt2 infers potential metagenomic functions from 16S rRNA gene profiles, these KO patterns should be interpreted as predicted functional potentials rather than direct measurements of gene abundance or activity [39]. In the present study, rearing water showed the highest overall predicted KO abundance, whereas host-associated compartments displayed distinct functional tendencies: surface mucus (TB) had relatively higher siderophore-related potential, stomach contents (GAS) had relatively higher quorum sensing-related potential, and intestine (INT) retained higher core predicted chemical-defense-related potential (Figure 6). This compartmental pattern is consistent with the broader concept that sea urchin-associated microbiomes are structured across different host microhabitats, including digestive compartments, coelomic fluid, and external surfaces [2]. These patterns suggest that different microhabitats impose unique ecological pressures that may favor iron acquisition and microbial competition at the host surface and chemical communication within the digestive microenvironment.

The higher predicted siderophore-related KO potential in surface mucus is consistent with previous studies showing that siderophores can function not only as iron-acquisition molecules but also as mediators of microbial competition and host-associated interactions [34,40]. This is particularly relevant for surface mucus, because marine microbial surface colonization and biofilm formation are strongly influenced by competitive interactions, nutrient acquisition, and chemically mediated cell–surface processes [41]. In our study, *Sulfurovaceae*/*Sulfurovum* were prominent in surface mucus, supporting the idea that this compartment may represent a chemically structured interface rather than a passive surface (Figure 3). However, siderophore production, iron competition, biofilm formation, and antimicrobial activity were not directly measured in this study. Therefore, the higher siderophore-related KO potential in surface mucus should be interpreted as a predicted functional signal rather than direct evidence of enhanced chemical-defense activity or secondary metabolite production.

Similarly, the enrichment of quorum sensing-related KOs in stomach contents is consistent with the role of quorum sensing in regulating bacterial communication, biofilm formation, secretion systems, and coordinated population-level responses [10,30]. Because stomach contents are influenced by dietary input and locally enriched organic substrates, this digestive compartment may provide conditions that favor microbial communication and interaction. Previous studies on sea urchin gut microbiomes have also shown that digestive compartments contain selective microbial assemblages and predicted metabolic functions distinct from environmental sources, supporting the idea that gut-associated microhabitats may harbor compartment-specific microbial functional potential [4,15]. However, the present study did not directly measure quorum sensing activity, secretion-system activity, biosynthetic gene clusters, metabolites, or antimicrobial effects.

Together, these results suggest a compartment-specific mosaic of predicted chemical-defense-related functional potential across cultured *H. pulcherrimus* microhabitats. Surface mucus and stomach contents may therefore be considered candidate compartments for future validation because they showed relatively higher predicted siderophore-related and quorum sensing-related KO potentials among host-associated compartments, respectively. However, they should not be described as confirmed priority targets for natural-product discovery or bioactive microorganism screening based on the current 16S rRNA gene and PICRUSt2-derived data alone.

### 3.7. Potential Aquaculture Relevance of Compartment-Specific Microbiomes in Cultured H. pulcherrimus

From an aquaculture perspective, compartment-specific microbiomes may be relevant to host health, microbial colonization resistance, and environmental microbial transmission in cultured *H. pulcherrimus*. The rearing water contained the highest microbial richness and a large number of unique ASVs, indicating that the aquaculture environment may act as an important microbial reservoir for host-associated compartments. In contrast, surface mucus, coelomic fluid, stomach contents, and intestine contained more restricted microbial assemblages, suggesting potential host- or microhabitat-associated filtering. Several differentially abundant taxa, including *Vibrio*, *Arcobacter*, *Sulfurovum*, and *Colwellia*, may be relevant to future studies of aquaculture health and microbial risk assessment. However, their detection in this study should not be interpreted as direct evidence of disease because pathogen isolation, virulence assays, and challenge experiments were not performed. These results provide candidate microbial indicators and compartments for future health monitoring and microbiome-based management of sea urchin aquaculture.

### 3.8. Limitations and Future Directions

Several limitations should be acknowledged. First, this study was based on 16S rRNA gene amplicon sequencing and PICRUSt2-based KO prediction; therefore, the predicted chemical-defense-related functions do not provide direct evidence of metabolite production, antibiotic activity, siderophore production, or biosynthetic gene clusters. Second, culture-dependent isolation of dominant bacterial taxa was not performed, and the physiological traits or bioactive potential of the major bacteria remain to be validated. Third, this study focused only on aquaculture-reared, apparently healthy *H. pulcherrimus* with similar body sizes, and did not include wild/free-living specimens or different age groups. Fourth, disease challenge experiments and pathogen verification were not conducted; therefore, the potential relevance of the microbiome to aquaculture disease should be interpreted as hypothesis-generating. Fifth, this pooling strategy reduced the ability to evaluate inter-individual microbiome variation, which should be addressed in future studies using individual-level sampling. Sixth, the differential abundance analyses also have statistical limitations. Kruskal–Wallis and Wilcoxon rank-sum tests were used as exploratory non-parametric approaches, but they do not fully account for the compositional and zero-inflated nature of microbiome relative abundance data. Therefore, the differentially abundant taxa and predicted KOs identified in this study should be interpreted as candidate patterns rather than definitive biomarkers or confirmed functional contributors. Seventh, differences among sample matrices may also have contributed to the observed microbiome patterns. Rearing water, surface mucus, coelomic fluid, stomach contents, and intestine differ substantially in biomass, matrix composition, inhibitor content, host-derived material, and environmental exposure. These matrix-related differences may influence DNA extraction efficiency, PCR amplification, and the detection of low-abundance taxa. Therefore, the observed compartmental differentiation likely reflects both biological microhabitat selection and matrix-associated methodological effects. Future studies should integrate culture-dependent screening, metagenomics, metabolomics, siderophore and antimicrobial assays, biosynthetic gene cluster analysis, wild-versus-cultured comparisons, and age-resolved sampling to validate the ecological and predicted chemical-defense-related roles of *H. pulcherrimus*-associated microbiota. Optimized extraction protocols, mock communities, extraction controls, and matrix-specific validation strategies will help distinguish true biological differences from potential methodological effects.

## 4. Materials and Methods

### 4.1. Sample Collection

*H. pulcherrimus* individuals were cultured under relatively stable aquaculture conditions before sampling. The water temperature was maintained at 19–22 °C, salinity at approximately 28, and pH at 8.0. Dissolved oxygen was kept at ≥5 mg/L, while ammonia nitrogen and reactive phosphate were maintained at ≤0.2 mg/L and ≤0.05 mg/L, respectively. Fresh kelp was used as the main feed. Samples were collected from cultured *H. pulcherrimus* and their aquaculture environment under sterile conditions. Five sample types were included: rearing water (AW), surface mucus (TB), coelomic fluid (BCF), stomach contents (GAS), and intestine (INT). The aquaculture system was divided into different sampling areas, and three apparently healthy sea urchins with similar body sizes were randomly selected from each area. Samples from the three individuals within each area were pooled by compartment, immediately stored under low-temperature conditions, and transported to the laboratory for DNA extraction. For each sample type, three biological replicates were analyzed, resulting in 15 samples in total: AW1–AW3, TB1–TB3, BCF1–BCF3, GAS1–GAS3, and INT1–INT3. For host-associated compartments, each biological replicate consisted of pooled material from three sea urchins collected within the same sampling area. Thus, a total of nine sea urchins contributed to each host-associated sample type. This pooling strategy was used to obtain sufficient material and to reduce individual-level variation within each sampling area. See the Appendix A for details.

### 4.2. DNA Extraction and Quality Inspection

Genomic DNA was extracted using the DNeasy PowerSoil Pro Kit (QIAGEN GmbH, Hilden, Germany) following the manufacturer’s protocol. DNA concentration and quality were measured using a Qubit 3.0 Fluorometer (Life Technologies Corporation, Carlsbad, CA, USA), agarose gel electrophoresis, and NanoDrop ND-2000 spectrophotometer (Thermo Fisher Scientific, Wilmington, DE, USA).

### 4.3. Amplification of the 16S rRNA Gene

The V4 region of bacterial and archaeal 16S rRNA genes was amplified using the universal primer pair 515FmodF/806R. The forward primer 515FmodF was 515F-Y (5′-GTGYCAGCMGCCGCGGTAA-3′), and the reverse primer was a degenerate/modified 806R variant (5′-GGACTACHVGGGTWTCTAAT-3′). Degenerate bases were included in these primers to improve coverage of bacterial and archaeal 16S rRNA gene sequences and to reduce known primer biases in marine microbial community surveys [42]. PCR reactions were prepared with DNA template, primers, FastPfu buffer, polymerase, BSA, and dNTPs, with amplification performed for 29 cycles. Thermal cycling conditions included initial denaturation, followed by cycles of denaturation, annealing, and extension, and a final extension step. PCR products were verified for expected size and purity before sequencing. Extraction blanks and PCR negative controls were included to monitor potential contamination during DNA extraction and amplification.

### 4.4. Illumina Sequencing and Bioinformatic Analysis

Purified amplicons were pooled in equimolar concentrations and sequenced on the Illumina MiSeq PE300 platform. Raw reads were processed using QIIME 2 (version 2024.10), including demultiplexing, primer trimming, quality filtering, denoising, merging, and chimera removal using the DADA2 plugin (in QIIME 2). Non-singleton ASVs were aligned with MAFFT (version 7.520), and phylogenetic trees were constructed with FastTree2 (version 2.1.11). Taxonomy was assigned using the classify-sklearn naïve Bayes classifier against the SILVA 138 database (version 138.1). ASVs assigned to chloroplasts, mitochondria, eukaryotes, or other non-target lineages were removed before downstream analyses [43,44,45,46,47,48,49].

### 4.5. Network Analysis

Co-occurrence networks were constructed using the Molecular Ecological Network Analysis Pipeline (MENAP) with Random Matrix Theory (RMT)-based threshold determination [50]. Prior to network construction, low-abundance and low-prevalence taxa were filtered to reduce spurious associations. Pairwise Spearman correlations were calculated, and only robust and statistically significant associations were retained according to the selected correlation threshold and FDR-adjusted *p* value < 0.05. Network visualization and topological analyses were performed using the igraph (version 2.1.1) package in R (version 4.3.2). Zi–Pi analysis was used to classify microbial nodes into peripherals, connectors, module hubs, and network hubs. Because the network was based on correlation analysis, the resulting edges were interpreted as statistical co-occurrence patterns rather than direct ecological interactions.

### 4.6. KO-Based Prediction of Chemical-Defense-Related Functions

Chemical-defense-related KOs were selected according to KEGG annotations and their relevance to microbial interaction, siderophore-related iron acquisition, quorum sensing, bacterial competition, and secretion-system-associated processes. Prediction reliability was evaluated using the weighted nearest sequenced taxon index (NSTI), where lower values indicate closer representation of ASVs by reference genomes and therefore greater confidence in functional prediction. The selected KOs were classified into four categories: core chemical-defense-related pathways, siderophore-related KOs, quorum sensing-related KOs, and bacterial competition/secretion-system-related KOs. Broad central metabolic pathways, housekeeping genes, general nutrient transporters, and pathways without a clear KEGG annotation or literature-supported connection to microbial interaction or chemical-defense-related hypotheses were excluded to avoid overinterpretation. The complete KO list, KEGG annotations, assigned categories, and classification rationale are provided in Supplementary Table S3. Functional prediction was performed using PICRUSt2 to evaluate the abundance of predicted chemical-defense-related KOs, including core pathways, siderophore-related functions, quorum sensing, and bacterial competition/secretion system-related functions [39]. Differences among sample types were tested using Kruskal–Wallis tests with FDR correction. Because these KO profiles were inferred from 16S rRNA gene data, they represent predicted functional potential rather than direct evidence of gene presence, gene expression, siderophore production, antibiotic biosynthesis, or bioactive compound activity.

### 4.7. Statistical Analysis and Visualization

Alpha diversity indices, including Chao1, Sobs, Shannon, and Coverage, were calculated to evaluate within-sample richness, diversity, and sequencing depth. Because the number of biological replicates per sample type was limited, Kruskal–Wallis tests were used as exploratory non-parametric comparisons of relative abundance rather than as definitive microbiome-specific differential abundance models. We acknowledge that microbiome relative abundance data are compositional and often zero-inflated; therefore, the results of these tests were interpreted cautiously as candidate compartment-associated patterns. When significant overall differences were detected, pairwise Wilcoxon rank-sum tests were used for post hoc comparisons. Beta diversity was assessed using Bray–Curtis dissimilarity based on ASV-level community composition and visualized using non-metric multidimensional scaling (NMDS). The stress value was used to evaluate the reliability of the ordination. Group-level differences in microbial community composition were tested using analysis of similarities (ANOSIM). UpSet diagrams and heatmaps were used to summarize shared, unique, and dominant microbial taxa across sample types [51,52,53]. Spearman correlation analysis and network analyses were conducted to evaluate statistical associations among microbial taxa. Correlation-based networks were interpreted as co-occurrence patterns rather than direct ecological interactions. Unless otherwise stated, statistical significance was defined as *p* < 0.05, and FDR-adjusted *p* values were used where multiple comparisons were performed [54].

## 5. Conclusions

This study revealed clear microbial community differentiation among rearing water, coelomic fluid, intestine, stomach contents, and surface mucus of cultured *H. pulcherrimus*. Rearing water harbored the highest microbial richness and the largest number of unique ASVs, indicating its role as a broad environmental microbial reservoir. In contrast, host-associated compartments showed distinct microbial compositions and a relatively high proportion of specialist taxa, suggesting strong microhabitat-specific selection. KO-based functional prediction further suggested that predicted chemical-defense-related microbial potential differed among compartments. Surface mucus and stomach contents showed relatively higher predicted siderophore-related and quorum sensing-related potentials among host-associated compartments, respectively. These findings do not provide direct biochemical evidence of chemical defense, but they identify candidate compartments, microbial groups, and functional categories for future metagenomic, metabolomic, and culture-dependent validation.

## Data Availability

The original contributions presented in the study are included in the article/Supplementary Materials. The raw sequence data are available in the Genome Sequence Archive (Genomics, Proteomics & Bioinformatics 2021) at the National Genomics Data Center (Nucleic Acids Res 2022), China National Center for Bioinformation/Beijing Institute of Genomics, Chinese Academy of Sciences (accession codes: CRA043344), and can be accessed at https://ngdc.cncb.ac.cn/gsa accessed on 15 May 2026. Data availability for peer review: CRA043344 (https://ngdc.cncb.ac.cn/gsub/submit/gsa/subCRA070978 accessed on 15 May 2026). Further inquiries can be directed to the corresponding author.

## Supplementary Materials

The following supporting information can be downloaded at: https://www.mdpi.com/article/10.3390/md24070243/s1, Figure S1: Sequencing depth and alpha diversity of microbial communities across sample types (A) Rarefaction curve for Chao1; (B) Rarefaction curve for Shannon; (C) Rarefaction curve for Coverage; (D) Alpha diversity estimators for Chao1; (E) Alpha diversity estimators for Shannon; (F) Alpha diversity estimators for Coverage (AW, rearing water; TB, surface mucus; BCF, coelomic fluid; GAS, stomach contents; INT, intestine); Figure S2: UpSet analysis of shared and unique ASVs among sample types (AW, rearing water; TB, surface mucus; BCF, coelomic fluid; GAS, stomach contents; INT, intestine). Each sample type contained three biological replicates. For host-associated compartments, each biological replicate represented pooled material from three sea urchins collected within the same sampling area; Figure S3: UpSet analysis of shared and unique microbial phyla among sample types (AW, rearing water; TB, surface mucus; BCF, coelomic fluid; GAS, stomach contents; INT, intestine). Each sample type contained three biological replicates. For host-associated compartments, each biological replicate represented pooled material from three sea urchins collected within the same sampling area; Figure S4: UpSet analysis of shared and unique microbial classes among sample types (AW, rearing water; TB, surface mucus; BCF, coelomic fluid; GAS, stomach contents; INT, intestine). Each sample type contained three biological replicates. For host-associated compartments, each biological replicate represented pooled material from three sea urchins collected within the same sampling area; Figure S5: UpSet analysis of shared and unique microbial families among sample types (AW, rearing water; TB, surface mucus; BCF, coelomic fluid; GAS, stomach contents; INT, intestine). Each sample type contained three biological replicates. For host-associated compartments, each biological replicate represented pooled material from three sea urchins collected within the same sampling area; Figure S6: UpSet analysis of shared and unique microbial genera among sample types (AW, rearing water; TB, surface mucus; BCF, coelomic fluid; GAS, stomach contents; INT, intestine). Each sample type contained three biological replicates. For host-associated compartments, each biological replicate represented pooled material from three sea urchins collected within the same sampling area; Figure S7: Class-level taxonomic composition of microbial communities across sample types (AW, rearing water; TB, surface mucus; BCF, coelomic fluid; GAS, stomach contents; INT, intestine). Each sample type contained three biological replicates. For host-associated compartments, each biological replicate represented pooled material from three sea urchins collected within the same sampling area; Figure S8: Order-level taxonomic composition of microbial communities across sample types (AW, rearing water; TB, surface mucus; BCF, coelomic fluid; GAS, stomach contents; INT, intestine). Each sample type contained three biological replicates. For host-associated compartments, each biological replicate represented pooled material from three sea urchins collected within the same sampling area; Figure S9: Family-level taxonomic composition of microbial communities across sample types (AW, rearing water; TB, surface mucus; BCF, coelomic fluid; GAS, stomach contents; INT, intestine). Each sample type contained three biological replicates. For host-associated compartments, each biological replicate represented pooled material from three sea urchins collected within the same sampling area; Figure S10: Genus-level taxonomic composition of microbial communities across sample types (AW, rearing water; TB, surface mucus; BCF, coelomic fluid; GAS, stomach contents; INT, intestine). Each sample type contained three biological replicates. For host-associated compartments, each biological replicate represented pooled material from three sea urchins collected within the same sampling area; Table S1: Alpha diversity indices of microbial communities across rearing water and *H. pulcherrimus*-associated compartments; Table S2: Topological roles of microbial taxa in the co-occurrence network based on Zi–Pi analysis; Table S3: Predicted abundance matrix of predicted chemical-defense-related KOs across rearing water and *H. pulcherrimus*-associated compartments; Table S4: Weighted of Nearest Sequenced Taxon Index (NSTI).

## Appendix A. Detailed Protocols

### Appendix A.1. Sample Collection

Samples were collected from cultured *H. pulcherrimus* and their aquaculture environment under sterile conditions. Five sample types were included in this study: rearing water (AW), surface mucus (TB), coelomic fluid (BCF), stomach contents (GAS), and intestine (INT). To reduce spatial sampling bias, the aquaculture system was divided into different sampling areas. From each sampling area, three apparently healthy sea urchins with similar body sizes and no visible external lesions were randomly selected and pooled to generate one biological replicate for each host-associated sample type.

Samples with the same numerical identifier represented the same sampling area rather than the same individual sea urchin. For example, TB1, BCF1, GAS1, and INT1 were collected from three randomly selected sea urchins within sampling area 1, and each sample represented a pooled compartment-specific sample from these three individuals. Similarly, TB2, BCF2, GAS2, and INT2 were collected from three randomly selected sea urchins within sampling area 2. This sampling strategy was designed to reduce individual-level variation and better represent the microbial characteristics of each sampling area.

Rearing water samples were collected from the corresponding sampling areas as environmental microbial references. During sampling, rearing water was collected first to avoid disturbance caused by animal handling. Surface mucus was then gently collected from the external body surface of each selected sea urchin using sterile swabs or sterile sampling tools, and mucus samples from three individuals within the same sampling area were pooled as one TB sample. Coelomic fluid was withdrawn using sterile syringes, and coelomic fluid from three individuals within the same sampling area was pooled as one BCF sample. The sea urchins were then dissected aseptically, and stomach contents and intestine were collected separately. For each sampling area, stomach contents and intestinal samples from three randomly selected individuals were pooled to generate one GAS and one INT sample, respectively. Sampling tools were replaced or sterilized between sample types and sampling areas to minimize cross-contamination. All samples were immediately placed under low-temperature conditions and transported to the laboratory for DNA extraction.

### Appendix A.2. KO-Based Functional Prediction

Functional profiles were predicted using PICRUSt2 based on the ASV table. KO annotations related to chemical defense were extracted and classified into four functional groups: core predicted chemical-defense pathways, siderophore-related functions, quorum sensing-related functions, and bacterial competition- and secretion system-related functions. Predicted KO abundances were compared across rearing water, coelomic fluid, intestine, stomach contents, and surface mucus. Statistical significance was evaluated using Kruskal–Wallis tests, with FDR correction applied for multiple comparisons.

### Appendix A.3. Statistical Analysis and Visualization

Alpha diversity metrics, including Chao1, Sobs, Shannon, and Coverage indices, were calculated to assess within-sample microbial richness, diversity, and sequencing depth. Differences among sample types were evaluated using Kruskal–Wallis tests, followed by pairwise Wilcoxon rank-sum tests when significant differences were detected. Beta diversity was assessed using Bray–Curtis dissimilarity and visualized using NMDS ordination. Group-level differences in community composition were evaluated with ANOSIM using 999 permutations. Shared and unique taxa were visualized with Upset diagrams, and heatmaps of dominant ASVs or genera were generated from normalized abundance profiles. Co-occurrence networks were constructed using dominant taxa, and topological properties, including Zi–Pi values, were calculated to assess the roles of taxa within inferred networks. All statistical analyses and visualizations were performed in R using the vegan (version 2.6.8), ggplot2 (version 3.6.1), and igraph (version 2.1.1) packages. Co-occurrence patterns were interpreted as statistical associations rather than direct ecological interactions.
