# Supplementary material for: Microbial Community Differentiation and Predicted Chemical-Defense-Related Functional Potential Across Distinct Microhabitats of Cultured Hemicentrotus pulcherrimus
Source: Mar Drugs. 2026 Jul 10;24(7):243. doi: 10.3390/md24070243 (PMC13413085; doi:10.3390/md24070243)
Supplement: Supplementary file 1 [file marinedrugs-24-00243-s001.zip › Supplementary Figure.pdf]

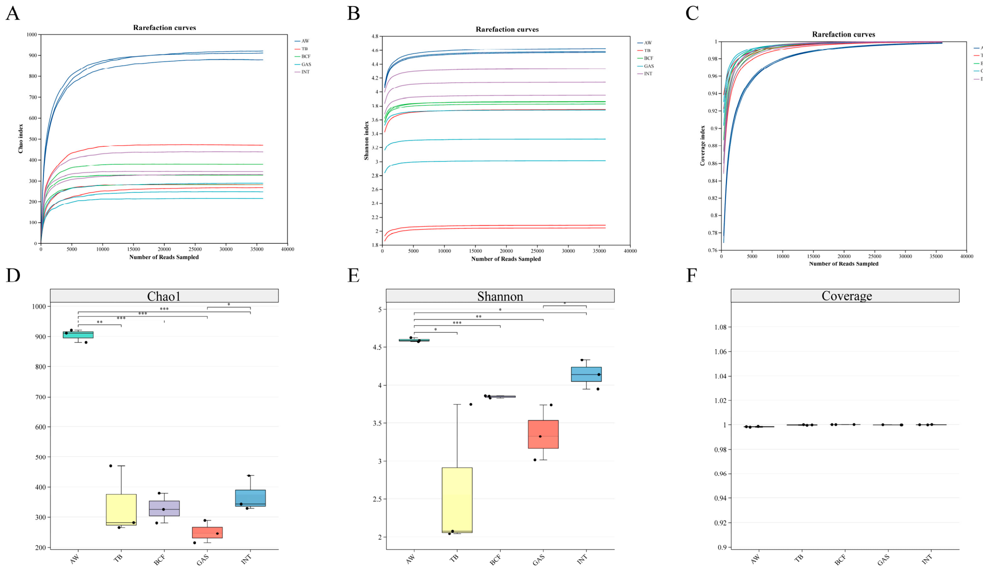

Supplementary Figure S1. Sequencing depth and alpha diversity of microbial communities across sample types (A) Rarefaction curve for Chao1; (B) Rarefaction curve for Shannon; (C) Rarefaction curve for Coverage; (D) Alpha diversity estimators for Chao1; (E) Alpha diversity estimators for Shannon; (F) Alpha diversity estimators for Coverage (AW, rearing water; TB, surface mucus; BCF, coelomic fluid; GAS, stomach contents; INT, intestine).

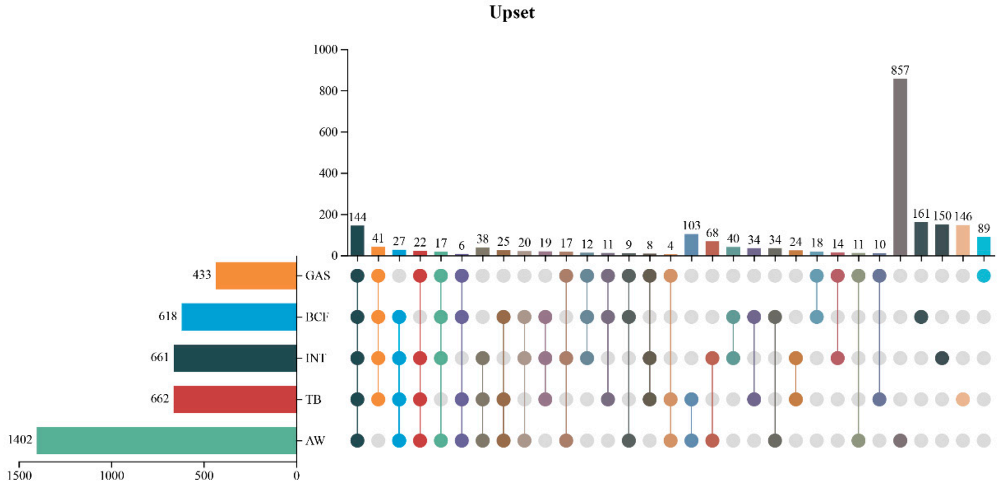

Supplementary Figure S2. UpSet analysis of shared and unique ASVs among sample types (AW, rearing water; TB, surface mucus; BCF, coelomic fluid; GAS, stomach contents; INT, intestine). Each sample type contained three biological replicates. For host-associated compartments, each biological replicate represented pooled material from three sea urchins collected within the same sampling area.

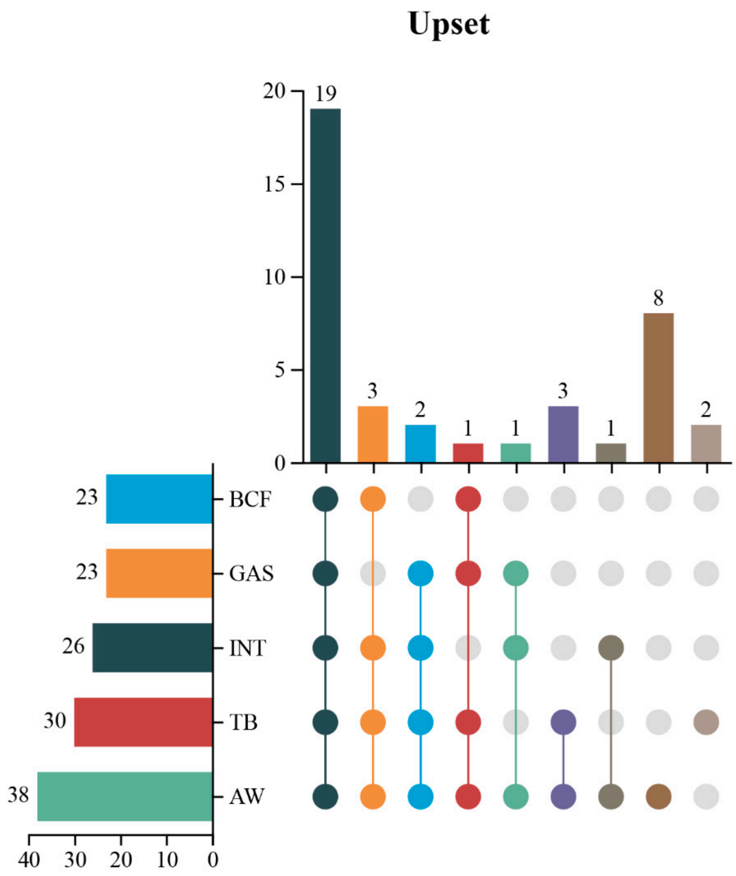

Supplementary Figure S3. UpSet analysis of shared and unique microbial phyla among sample types (AW, rearing water; TB, surface mucus; BCF, coelomic fluid; GAS, stomach contents; INT, intestine). Each sample type contained three biological replicates. For host-associated compartments, each biological replicate represented pooled material from three sea urchins collected within the same sampling area.

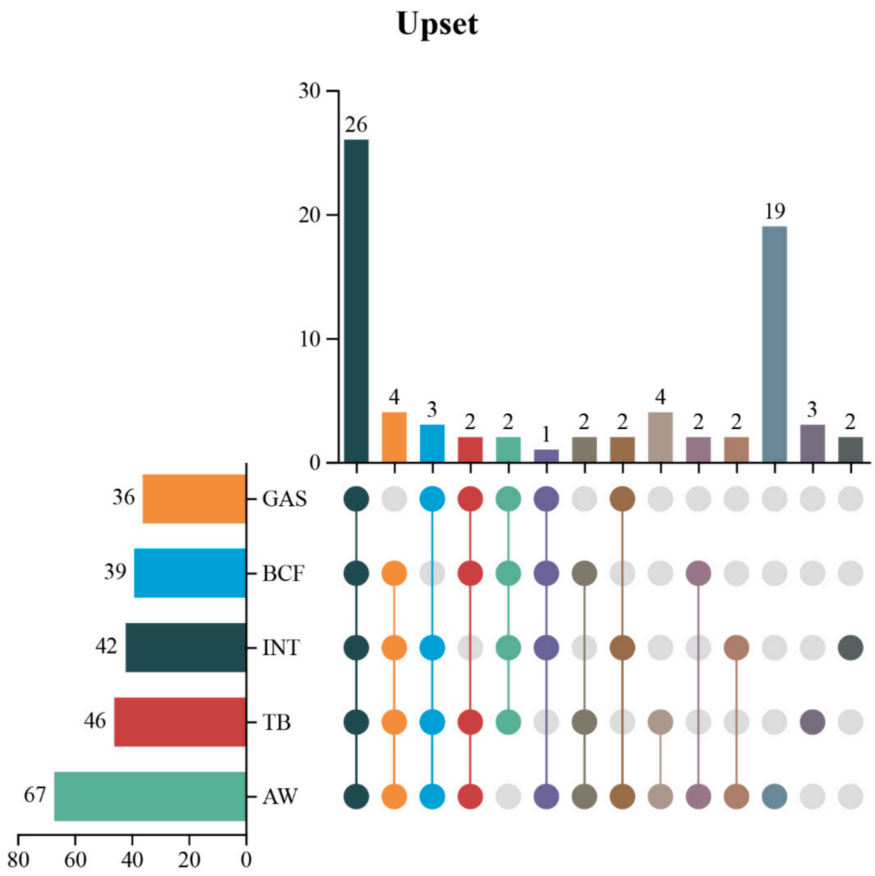

Supplementary Figure S4. UpSet analysis of shared and unique microbial classes among sample types (AW, rearing water; TB, surface mucus; BCF, coelomic fluid; GAS, stomach contents; INT, intestine). Each sample type contained three biological replicates. For host-associated compartments, each biological replicate represented pooled material from three sea urchins collected within the same sampling area.

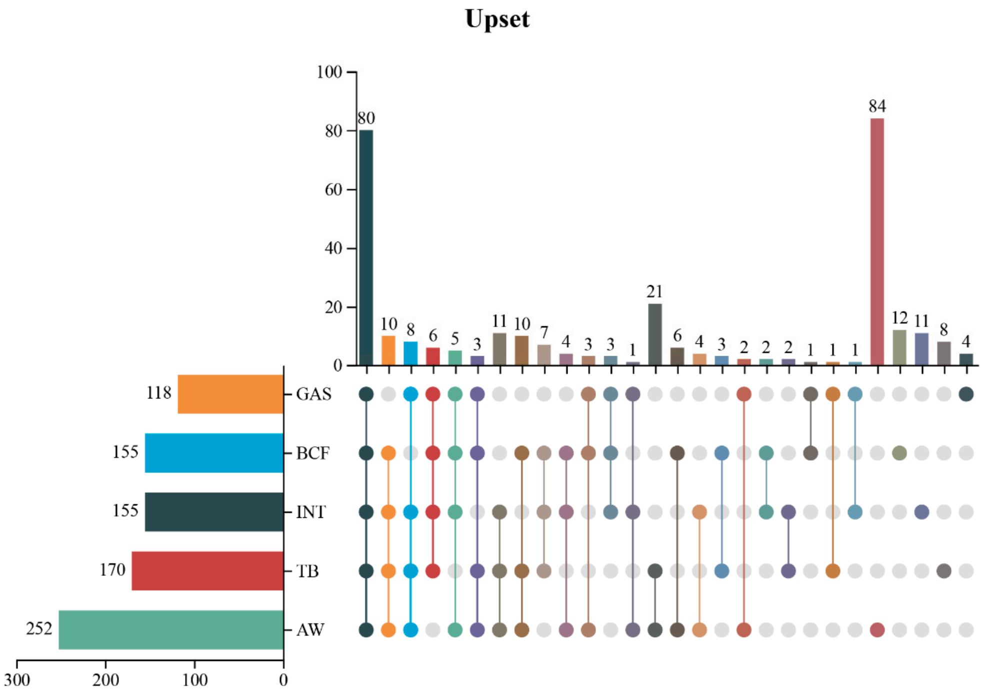

Supplementary Figure S5. UpSet analysis of shared and unique microbial families among sample types (AW, rearing water; TB, surface mucus; BCF, coelomic fluid; GAS, stomach contents; INT, intestine). Each sample type contained three biological replicates. For host-associated compartments, each biological replicate represented pooled material from three sea urchins collected within the same sampling area.

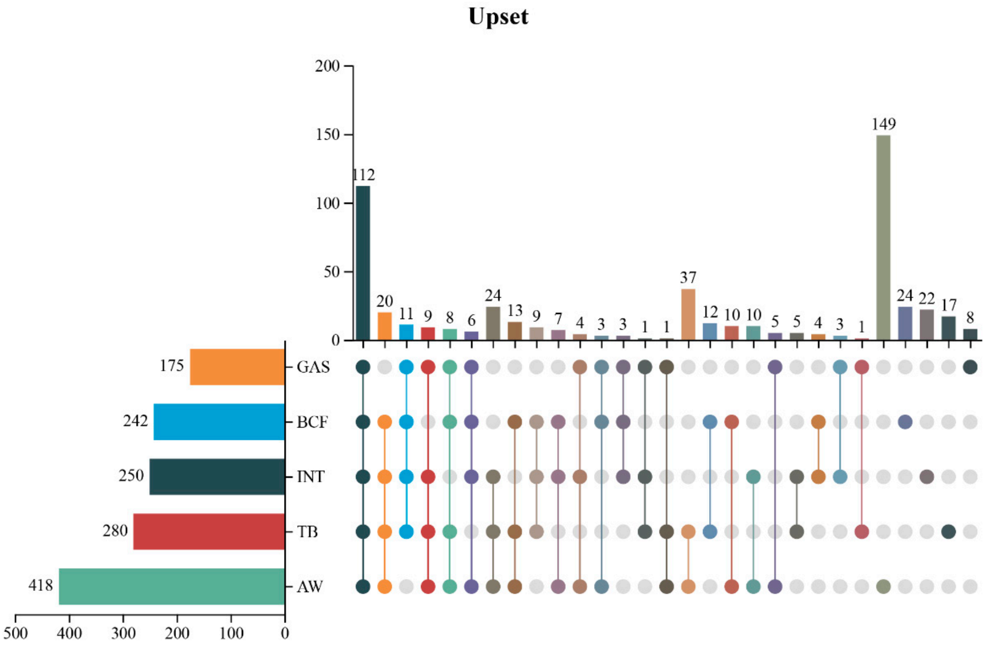

Supplementary Figure S6. UpSet analysis of shared and unique microbial genera among sample types (AW, rearing water; TB, surface mucus; BCF, coelomic fluid; GAS, stomach contents; INT, intestine). Each sample type contained three biological replicates. For host-associated compartments, each biological replicate represented pooled material from three sea urchins collected within the same sampling area.

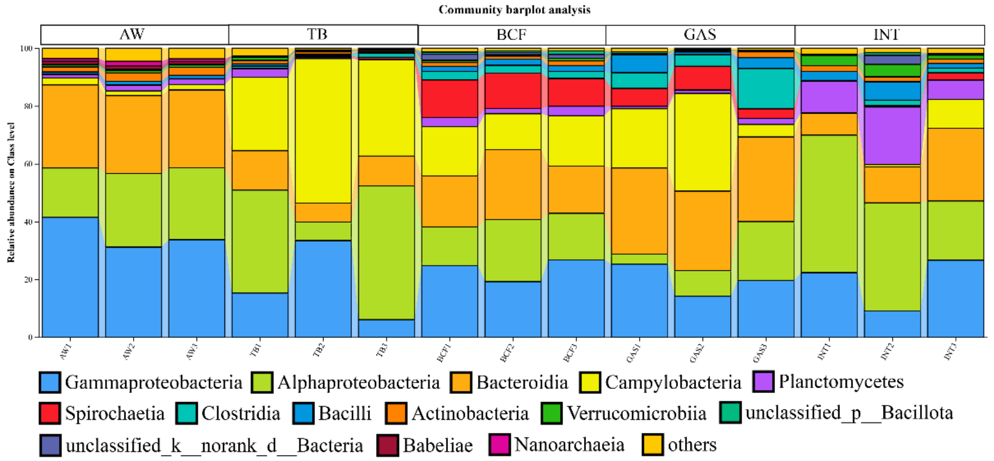

Supplementary Figure S7. Class-level taxonomic composition of microbial communities across sample types (AW, rearing water; TB, surface mucus; BCF, coelomic fluid; GAS, stomach contents; INT, intestine). Each sample type contained three biological replicates. For host-associated compartments, each biological replicate represented pooled material from three sea urchins collected within the same sampling area.

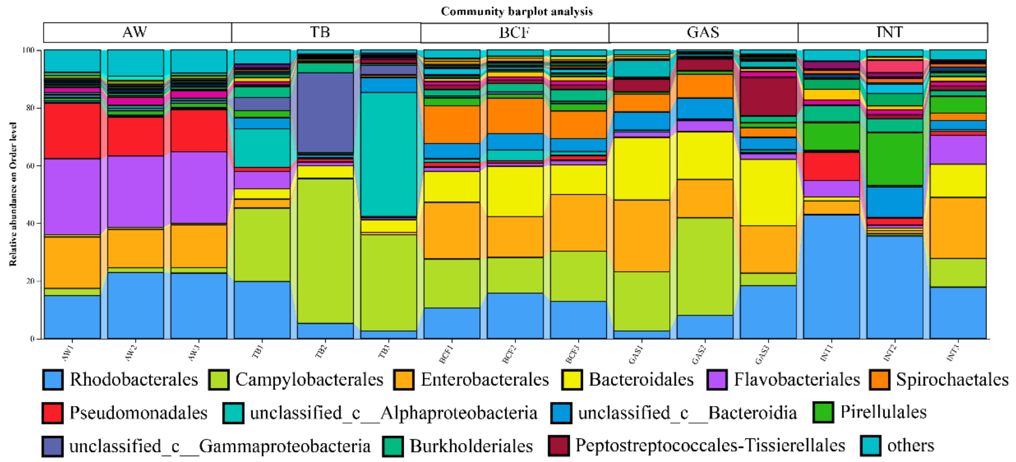

Supplementary Figure S8. Order-level taxonomic composition of microbial communities across sample types (AW, rearing water; TB, surface mucus; BCF, coelomic fluid; GAS, stomach contents; INT, intestine). Each sample type contained three biological replicates. For host-associated compartments, each biological replicate represented pooled material from three sea urchins collected within the same sampling area.

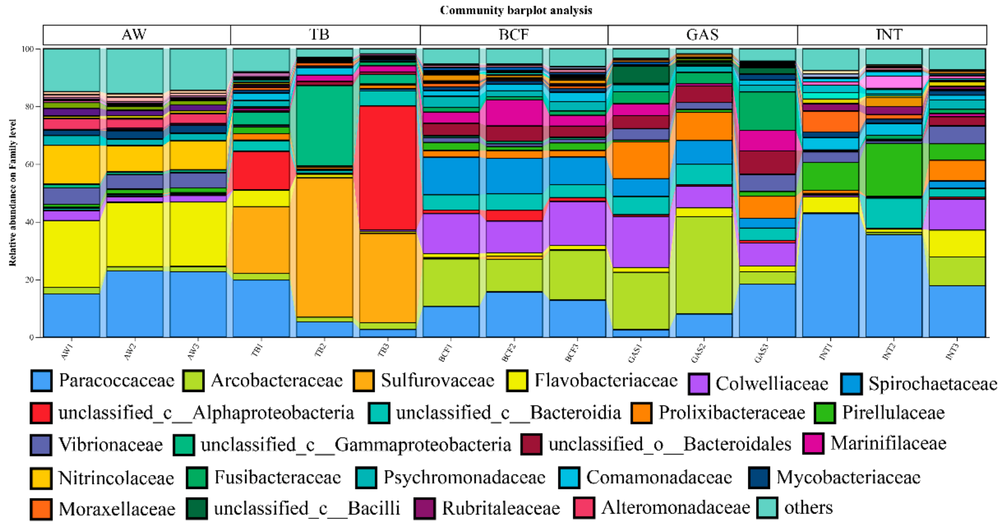

Supplementary Figure S9. Family-level taxonomic composition of microbial communities across sample types (AW, rearing water; TB, surface mucus; BCF, coelomic fluid; GAS, stomach contents; INT, intestine). Each sample type contained three biological replicates. For host-associated compartments, each biological replicate represented pooled material from three sea urchins collected within the same sampling area.

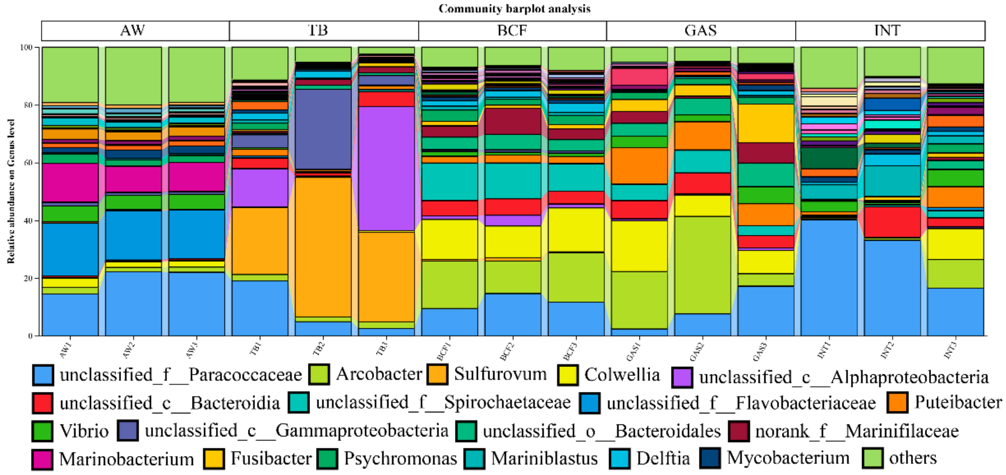

Supplementary Figure S10. Genus-level taxonomic composition of microbial communities across sample types (AW, rearing water; TB, surface mucus; BCF, coelomic fluid; GAS, stomach contents; INT, intestine). Each sample type contained three biological replicates. For host-associated compartments, each biological replicate represented pooled material from three sea urchins collected within the same sampling area.
